# 

**Published:** 1924-10

**Authors:** 


					xW//? ? ?
CONTENTS. Page
The Long Fox Lecture: The Conditioned Response.
By C. Lloyd
Morgan, LL.D., D.Sc., F.R.S.
165
AuriCuiar Failure.
By Carey F. Coombs, M.D., F.R.C.P. Lond.,
and C. E. K. Herapath, M.C., M.D. Lond.
i8o
Few Suggestions in General Surgery.
By C. F. Walters,
FR.C.S.
Reviews of Books
The F*rr Fund
In Mem
oriam.
Lemuel Matthews Griffiths, M.R.C.S., L.R.C.P. ...
213
Editorial Notes
2I4
The Lib
rary of the Bristol Medico-Chirurgical Society  215
*~?csi|
Medical Notes
216

				

